# Body posture can modulate liver stiffness measured by transient elastography: a prospective observational study

**DOI:** 10.1186/s12876-024-03473-8

**Published:** 2024-10-31

**Authors:** Zi-Hao Huang, Miao-Qin Deng, Yangmin Lin, Chen-Hui Ye, Ming-Hua Zheng, Yong-Ping Zheng

**Affiliations:** 1 Department of Biomedical Engineering, The Hong Kong Polytechnic University, Hong Kong, China; 2 MAFLD Research Center, Department of Hepatology, The First Affiliated Hospital of Wenzhou Medical University, Wenzhou, China; 3 Key Laboratory of Diagnosis and Treatment for the Development of Chronic Liver Disease in Zhejiang Province, Wenzhou, China; 4 Research Institute for Smart Ageing, The Hong Kong Polytechnic University, Hong Kong, China

**Keywords:** Body position, Measuring posture, Patient positioning, Liver fibrosis, Liver stiffness measurement, Ultrasound elastography

## Abstract

**Background:**

Non-invasive measurement of liver stiffness (LS), traditionally performed in the supine position, has been established to assess liver fibrosis. However, fibrosis degree is not the sole determinant of LS, necessitating the identification of relevant confounders. One often-overlooked factor is body posture, and it remains unclear whether normal daily postures interfere with LS irrespective of fibrosis. A prospective two-group comparison study was conducted to investigate the relationship between posture and LS.

**Methods:**

Sixty-two adults participated, divided into two groups: patients with chronic liver disease and healthy controls. Both groups were assessed using transient elastography (TE) under the supine, seated, and standing postures. Randomization was applied to the order of the two upright postures. A two-way mixed ANOVA was conducted to assess the posture-dependence of LS and its variations between two groups.

**Results:**

Results showed that posture differentially affected LS depending on the presence of liver fibrosis. In 31 healthy individuals (baseline LS range: 3.5–6.8 kPa), a transition from the supine (5.0 ± 1.0 kPa) to seated (5.7 ± 1.4 kPa; *p* = 0.036) or standing (6.2 ± 1.7 kPa; *p* = 0.002) positions increased LS, indicating liver stiffening. Conversely, in 31 patients with varying fibrosis stages (baseline LS range: 8.8–38.2 kPa), posture decreased LS from the supine (15.9 ± 7.3 kPa) to seated (13.8 ± 6.2 kPa; *p* < 0.001) or standing (13.9 ± 6.2 kPa; *p* = 0.001) positions. No significant difference in LS was observed between the seated and standing positions in both groups (control group: 5.7 vs. 6.2 kPa*, p* = 0.305; patient group: 13.8 vs. 13.9 kPa, *p* = 1). Additionally, different postures did not elicit significant changes in the success rate (supine, 98.6 ± 4%; seated, 97.6 ± 6%; standing, 99.1 ± 3%; *p* = 0.258) and IQR/median value (supine, 25 ± 8%; seated, 29 ± 15%; standing, 29 ± 12%; *p* = 0.117), implying no impact on both measurement feasibility and reliability.

**Conclusions:**

We demonstrated, for the first time, the feasibility of utilizing upright postures as an alternative measurement protocol for TE. We further unravel a previously unrecognized role of transitioning between different postures to assist the diagnosis of cirrhosis. The findings suggested that daily physiological activity of postural changes suffices to alter LS. Therefore, body positioning should be standardized and carefully considered when interpreting LS.

**Supplementary Information:**

The online version contains supplementary material available at 10.1186/s12876-024-03473-8.

## Introduction

Currently, liver biopsy is considered the gold standard for diagnosing the excessive accumulation of fibrous tissue in the liver. However, this invasive procedure carries the risk of life-threatening complications, and is limited by inter-pathologist variability and sampling error [1]. These drawbacks have prompted the widespread clinical adoption of transient elastography (TE), a technique that quantifies liver stiffness (LS) as a surrogate biomarker for liver fibrosis [2]. TE is a WHO-recommended ultrasound elastography technique for liver stiffness measurement (LSM) [3, 4], and has demonstrated excellent diagnostic accuracy in staging liver fibrosis through meta-analyses [5, 6].

LS below 8 kPa typically indicates the upper limit of normal, which is associated with a high negative predictive value for excluding clinically significant fibrosis (stage F ≥ 2) [7, 8], and possesses up to a 93% sensitivity for ruling out cirrhosis (stage F = 4) [9]. At the higher end of the LS spectrum, although increased LS (≥ 8 kPa) is widely recognized to primarily reflect a degree of fibrotic accumulation, various other factors can influence LS [4, 10, 11]. Certain pathophysiological conditions have been shown to falsely elevate LS in the absence of liver fibrosis. These confounding factors include amyloidosis [12]), necro-inflammation [13, 14], hepatic congestion [15, 16], cholestasis [17], respiration [18], food intake [19, 20], and blood pressure [21, 22]. For instance, two European studies have reported that liver damage from acute hepatitis can induce a reversible increase in LS to the level suggestive of cirrhosis (≥ 12.5 kPa), thus potentially misguiding the diagnosis of pre-existing cirrhosis [13, 14]. Likewise, Millonig et al. [15] identified cardiac insufficiency as another hemodynamic cause that can elevate LS irrespective of the fibrosis stage. Impaired venous drainage and intrahepatic blood stasis may be the primary reasons behind this false-positive result of LSM. Given the existing evidence, there is a pressing need to identify additional confounders for accurate interpretation of LS in the clinical setting.

Body posture may be one such factor to confound LS, as it also influences hepatic hemodynamics. Previous literature has documented morphological and hemodynamic responses of liver-associated vasculatures such as inferior vena cava (IVC) [23, 24], portal vein [25, 26] and aorta [23] to postural changes; however, the causative relationship between posture and LS has not been well explored. Along this line, this study aimed to assess the effect of the supine and upright positions (i.e., seated and standing) on LSM using TE. Given the previously established variations in anatomy and pathophysiology between healthy and cirrhotic livers [25, 27], we further hypothesized that the liver would respond differently to an upright posture depending on the degree of fibrosis. To test this hypothesis, we analysed the posture-induced LS differences in healthy individuals and patients across various fibrosis stages.

## Methods

### Study population

In this prospective study, we recruited two independent ‘LS groups’ via convenience sampling. Adults (≥ 18 years) who had been diagnosed with chronic liver disease (CLD) of any aetiology and clinically indicated for TE were eligible. Patients were recruited from The First Affiliated Hospital of Wenzhou Medical University and assigned to the patient group. In contrast, a cohort of healthy controls with no history of liver disease were recruited from another participating institute − The Hong Kong Polytechnic University to assemble the control group. Exclusion criteria for both groups included: (1) common contraindications for TE (e.g., ascites, pregnancy, or active cardiac implant); (2) previous liver transplantation; (3) known malignancy or other terminal disease; (4) age under 18 years; and (5) any ongoing confounding conditions known to cause interferences with LS (e.g., cholestasis, acute hepatitis, congestive heart failure, or portal hypertension in decompensated cirrhosis). Because the experiment involves procedures of postural changes, upright positioning, and the right arm raised to expose an acoustic window, individuals with limited shoulder mobility or balance disorders (e.g., Parkinson’s disease) were also deemed ineligible and thus excluded from this study.

A *priori* power analysis using the G*Power software (Heinrich-Heine-Universität Düsseldorf, Düsseldorf, Germany) projected a sample size of 56 under a two-way (3 × 2) mixed-model design, assuming a medium Cohen’s effect size of ƒ = 0.20 (η^2^_*p*_ = 0.04), 90% statistical power, and a two-tailed significance level of 0.05. The final target sample size was set at 62 to allow for 10% dropout. The Ethics Committees of both centers approved the study protocol. All subjects provided written informed consent. This observational, cross-sectional study was compliant with the STROBE criteria.

### Procedures

**Fig. 1:**
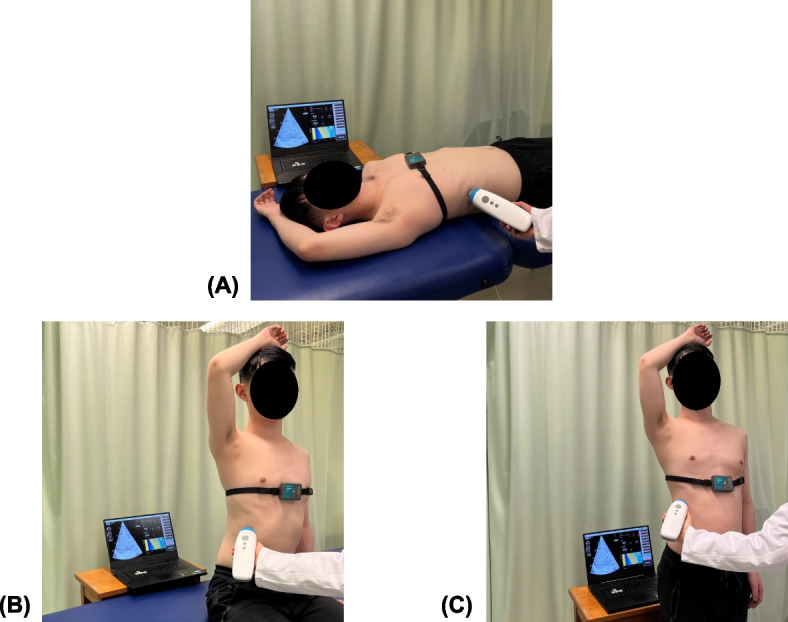
Experimental setup for TE-based LSM under three patient positioning techniques: (**A**) supine; (**B**) seated; (**C**) standing

Prior to LSM, demographic, anthropometric, and clinical data were collected according to standard protocols. Anthropometric tests included body weight, height, and waist circumference (WC) measurements. Subjects were classified as overweight or obese if their body mass index (BMI) exceeded 25 kg/m^2^ and 30 kg/m^2^, respectively. WC was measured at the midpoint between the lower rib margin and iliac crest, with the tape positioned around the body. Skin-liver capsule distance (SCD) was derived from abdominal sonography and quantified based on B-mode liver images. Liver fat was assessed quantitatively using controlled attenuation parameter (CAP) via the Fibroscan® system (Echosens, Paris, France). Diagnosis of steatosis was determined by a CAP score according to the published cut-off of ≥ 248 dB/m [28]. Medical history, including biopsy-proven liver fibrosis, hypertension, Type 2 diabetes mellitus (DM), and dyslipidaemia, was recorded. Type 2 DM was defined by a fasting plasma glucose level ≥ 7.0 mmol/L (126 mg/dL) or the use of glucose-lowering medications. Dyslipidaemia was defined by total cholesterol ≥ 5.2 mmol/L (200 mg/dL), low-density lipoprotein (LDL) cholesterol ≥ 3.4 mmol/L (130 mg/dL), high-density lipoprotein (HDL) cholesterol < 1.0 mmol/L (40 mg/dL) in men or < 1.3 mmol/L (50 mg/dL) in women, triglycerides ≥ 1.7 mmol/L (150 mg/dL), or the use of lipid-lowering medications. Hypertension was defined by a blood pressure of ≥ 140/90 mmHg or antihypertensive drug use. Presence of metabolic syndrome was defined according to the ethnic-specific criteria established by the International Diabetes Federation. To evaluate LS under three different postures, TE was conducted with the Liverscan® system (Eieling Technology Limited, Hong Kong, China) which enables simultaneous B-mode imaging of liver anatomy and LSM. The performance analysis of this B-mode guided TE has been previously detailed [29]. Briefly, the system measures the velocity of 50-Hz shear wave in the liver to estimate LS and has been experimentally validated using tissue-mimicking phantoms. Comparative studies against other liver elastography techniques, including conventional TE and two-dimensional shear wave elastography (2D-SWE) in human subjects, have demonstrated its reliability and validity of LSM in patients with CLD. All subjects fasted for a minimum of three hours before LSM to avoid a confounding post-prandial increase in LS. As previously described [2, 4], the standard patient positioning for TE examination requires maximal abduction of the right arm, which was consistently maintained across all three posture conditions. Each subject first underwent TE in the supine position as the baseline assessment, followed by measurements in the two upright positions: seated and standing (Fig. 1). The examination order of the latter two upright positions were randomized for subjects to eliminate the operator bias resulting from a learning effect. A five-minute interval between different posture conditions was allocated. Assisted by real-time B-mode, an investigator, who was specialized in radiography and had six years of TE experience, identified a representative portion of the right liver parenchyma free of large vasculatures, and selected it as the reference image. The corresponding measurement site was also marked on the subject’s skin surface. An attempt was made to collect exact 15 measurements under each of the three postures. Considering that the liver is a moving organ and not necessarily uniform, we took several measures to limit these confounding factors, i.e., respiratory motion and liver fibrosis heterogeneity. In addition, this study employed two different TE techniques (conventional vs. B-mode guided TE, see Supplementary Material) for measurements to validate LS values used for subsequent analyses.

### Outcome measures

**Fig. 2:**
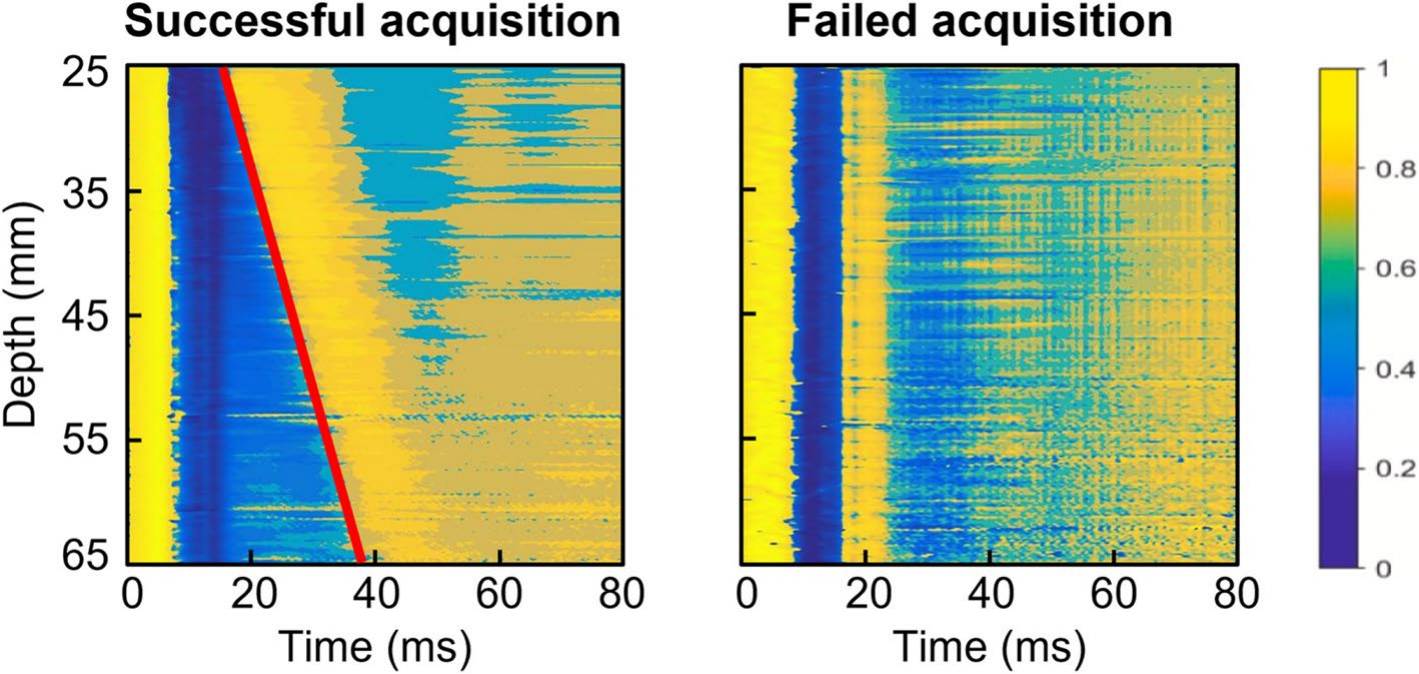
Representative example illustrating the manual classification outcomes for both a successful and a failed elastogram acquisition from the same subject. Classification system was devised on the basis of a binary criterion, with shear wave propagation in the liver indicating a successful LSM; and vice versa

LSM typically involves not only LS, but also its quality criteria [30]. The median LS of the first 10 successful acquisitions was calculated, and used in this study as the per-subject Young’s modulus of the liver for each posture condition. To allow easy implementation, simplified rounded LS of < 8 kPa, 8–13 kPa and ≥ 13 kPa were respectively considered normal, clinically significant fibrosis and cirrhosis [6, 7, 16], and were therefore adopted as cutoffs for the subsequent subgroup analyses. The interquartile range to median LS ratio (IQR/median) was determined as the evaluation metric of variability between the 10 LSMs. The success rate, defined as the ratio of successful acquisitions to the total of 15 measurement attempts, was also computed for each posture. This serves as the metric evaluating whether the feasibility of LSM would vary with posture. To achieve that, elastogram acquisition quality was qualitatively analyzed by an experienced human rater and determined on a binary scale (Fig. 2). Procedures of manual elastogram classification are detailed in the Supplementary Material.

### Statistical analyses

#### Descriptive statistics

Between-group comparisons were made using Fisher’s exact test for categorical data, and Mann–Whitney U test for continuous data. The significance level is set at 0.05.

#### Posture effect on LSM

A two-way mixed ANOVA was applied to examine the main effects of (a) within-subjects factor ‘posture’ (supine, seated, standing conditions) and (b) between-subjects factor ‘LS group’ (patient vs. control groups), and their interaction on LS. The success rate and IQR/median values between three posture conditions were compared separately using a one-way repeated measures ANOVA and non-parametric Friedman test, where appropriate.

The relationship between the LS measured in the benchmark supine position and the magnitude of change in LS from the supine to upright position (i.e., absolute value of stiffness-change [%]) was analysed using both Spearman’s correlation and simple linear regression.

#### Factor analysis

To assess the demographic, clinical and anthropometric factors that possibly influenced LS, we performed univariate analysis using the Spearman’s correlation test for continuous candidate variables, and the Mann–Whitney U test for categorical candidate variables. Variables that achieved statistical significance (*p* < 0.05) in the univariate analysis were included in a multivariate model. The multiple regression model was constructed with age, weight, BMI, SCD, WC, presence of overweight or obesity, Type 2 DM, and a history of biopsy-proven liver fibrosis as candidate covariates, and the supine, seated, and standing LS as outcome variables. All statistical analyses were performed using SPSS version 26.0 (IBM Corp., New York, USA) and GraphPad Prism 9 (GraphPad Software Inc., San Diego, USA).

## Results

### Demographics

Between October 2022 and June 2023, 32 patients and 31 controls were screened for eligibility in the study. A female patient with a BMI of 28 kg/m^2^ was excluded because LSM failure occurred repeatedly in all attempts. Overall, the median age of all subjects was 55 years, and 61% of them were male. The patient group consisted predominantly of those with metabolic dysfunction-associated steatotic liver disease (MASLD), among whom 45% had simple steatosis and 6% presented with concurrent hepatitis B virus (HBV) infection. Table 1 outlines the comparative analysis of the 62 subjects who were successfully enrolled into two groups.

**Table 1:** Baseline characteristics comparison between two groups

Variable	Patients (*n* = 31)	Controls (*n* = 31)	Total (*n* = 62)	*p*-value^¶^
•Demographics				
Male	71% (22)	52% (16)	61% (38)	0.192
Age, years	50 [17]	58 [14]	55 [18]	0.051
Overweight & obese	61% (19)	10% (3)	35% (22)	< 0.001*
Metabolic syndrome	29% (9)	10% (3)	19% (12)	0.106
• Anthropometrics				
BMI, kg/m^2^	25 [6]	23 [4]	24 [5]	< 0.001*
WC, cm	96 [21]	81 [15]	89 [18]	< 0.001*
SCD, mm	16 [7]	11 [7]	15 [8]	< 0.001*
• Liver disease etiology				
Viral (CHB, CHC)	35% (11)	-	-	-
MASLD	45% (14)	-	-	-
ALD	3% (1)	-	-	-
Coexistent CHB & ALD	10% (3)	-	-	-
Coexistent CHB & MASLD	6% (2)	-	-	-
• LSM in the supine position				
LS, kPa	13.1 [7]	4.6 [1]	7.8 [9]	< 0.001*
IQR/median, %	24 [13]	26 [13]	25 [12]	0.368
Success rate^§^, %	100 [0]/100 ± 0	100 [0]/97 ± 6	100 [0]/99 ± 4	0.005*
• LSM in the seated position				
LS, kPa	12.7 [7]	5.1 [2]	7.4 [8]	< 0.001*
IQR/median, %	24 [13]	30 [23]	25 [18]	0.208
Success rate^§^, %	100 [7]/96 ± 9	100 [0]/99 ± 2	100 [0]/98 ± 6	0.072
• LSM in the standing position				
LS, kPa	13.0 [7]	6.2 [2]	7.9 [7]	< 0.001*
IQR/median, %	26 [20]	30 [19]	28 [18]	0.559
Success rate^§^, %	100 [0]/99 ± 3	100 [0]/99 ± 3	100 [0]/99 ± 3	0.687

All data are median [IQR] or % (n), unless otherwise indicated

*ALD* Alcoholic liver disease, *BMI* Body mass index, *CHB* Chronic hepatitis B, *CHC* Chronic hepatitis C, *LS* Liver stiffness, *LSM* Liver stiffness measurement, *MASLD* Metabolic dysfunction-associated steatotic liver disease, *WC* Waist circumference, *SCD* Skin-liver capsule distance

^¶^Quantitative variables between groups were compared by Mann–Whitney U test; categorical variables were compared by Fisher’s exact test

^§^Data are presented as median [IQR] / mean ± standard deviation

^*^ *p* < 0.05

### Inter-posture LS difference comparison

#### Boxplots of LS vs

**Fig. 3:**
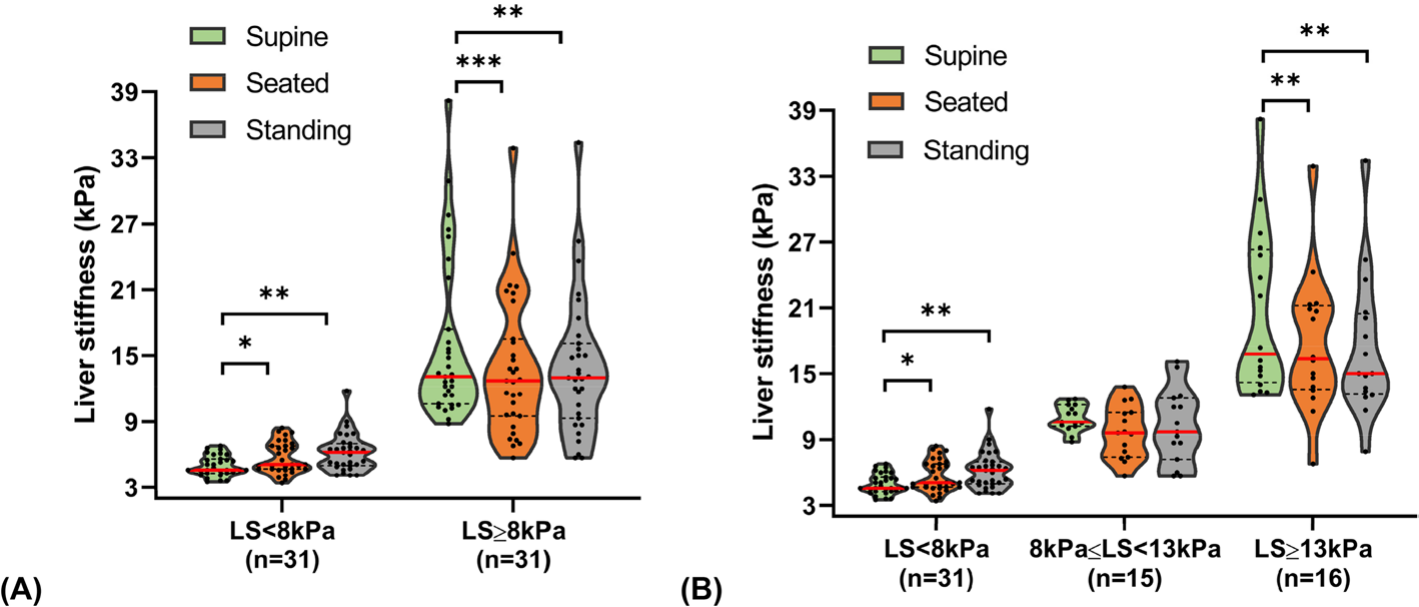
Effect of posture on LS between (**A**) two LS subgroups; (**B**) three LS subgroups (*p ≤ 0.05; **p ≤ 0.01; ***p ≤ 0.001; *p* > 0.05 otherwise)

Boxplots of LS vs. fibrosis stage stratified by posture are illustrated in Fig. 3. A two-way mixed ANOVA identified a statistically significant interaction between ‘posture’ and ‘LS group’ on LS, *F*(2,120) = 21.242, *p* < 0.001, partial η^2^ = 0.26. This implied that the effect of posture is dependent on the magnitude of LS itself. Therefore, two separate one-way repeated measures ANOVAs were run to examine simple main effects of ‘posture’ within each LS group. Of 31 controls with baseline LS < 8 kPa, significant differences in LS existed among three posture conditions, *F*(2,60) = 8.518, *p* < 0.001, partial η^2^ = 0.22. Post-hoc analyses with Bonferroni correction showed a statistically significant increase in LS from the supine to seated position (5.0 ± 1.0 vs. 5.7 ± 1.4 kPa; *p* = 0.036), and from the supine to standing position (5.0 ± 1.0 vs. 6.2 ± 1.7 kPa; *p* = 0.002), indicating that upright positioning generally causes the stiffening of healthy livers. However, LS did not significantly differ between these two upright positions (5.7 vs. 6.2 kPa, mean difference of 0.5 kPa; *p* = 0.305). Of note, we observed a greater magnitude of increase in LS in the standing position compared to the seated position (percentage change from supine: 24% vs. 14%).

In contrast, an opposite trend was noted in 31 patients with baseline LS > 8 kPa who were classified as having fibrosis, *F*(2,60) = 13.837, *p* < 0.001, partial η^2^ = 0.32. There was a statistically significant decrease in LS from the supine to seated position (15.9 ± 7.3 vs. 13.8 ± 6.2 kPa; *p* < 0.001), as well as from the supine to standing position (15.9 ± 7.3 vs. 13.9 ± 6.2 kPa; *p* = 0.001), but not between the seated and standing positions (13.8 vs. 13.9 kPa, mean difference of 0.03 kPa; *p* = 1.0). These post-hoc comparison results suggest liver softening in the upright postures among patients with fibrosis. The magnitude of decline in LS in the seated position was comparable to that in the standing position (percentage change from supine: 15% vs. 14%).

To determine the extent to which fibrosis can influence the response pattern of LS to postural changes, 31 patients were further divided into two fibrosis categories based on predefined cutoffs. Subgroup analyses were conducted according to the degree of liver fibrosis (i.e., no fibrosis vs. clinically significant fibrosis vs. cirrhosis). In cirrhotic patients with baseline LS > 13 kPa, LS declined markedly as the posture transitioned from the supine to seated position (20.5 ± 7.6 vs. 17.7 ± 6.3 kPa, percentage decrease: 16%; *p* = 0.005) and to standing position (20.5 ± 7.6 vs. 17.3 ± 6.4 kPa, percentage decrease: 18%; *p* < 0.001). Interestingly, LS remained fairly unchanged across all postures in patients with baseline LS of 8–13 kPa, indicative of less fibrosis (supine, 11.0 ± 1.2 kPa; seated, 9.7 ± 2.4 kPa; standing, 10.2 ± 3.4 kPa; *p* = 0.107).

An intraindividual analysis was further conducted within these three subgroups. The results indicate that the posture-induced changes in LS led to discordance of at least one predefined stage between the supine and upright positions. Among the healthy controls, five out of 31 (16%) shifted from no fibrosis (< 8 kPa) to a higher category. Of 31 patients, four (13%) shifted from cirrhosis (≥ 13 kPa) to a lower category, while nine (29%) with fibrosis (8–13 kPa) shifted to an adjacent category, i.e., either no fibrosis or cirrhosis.

##### Percentage change of stiffness between postures

**Fig. 4:**
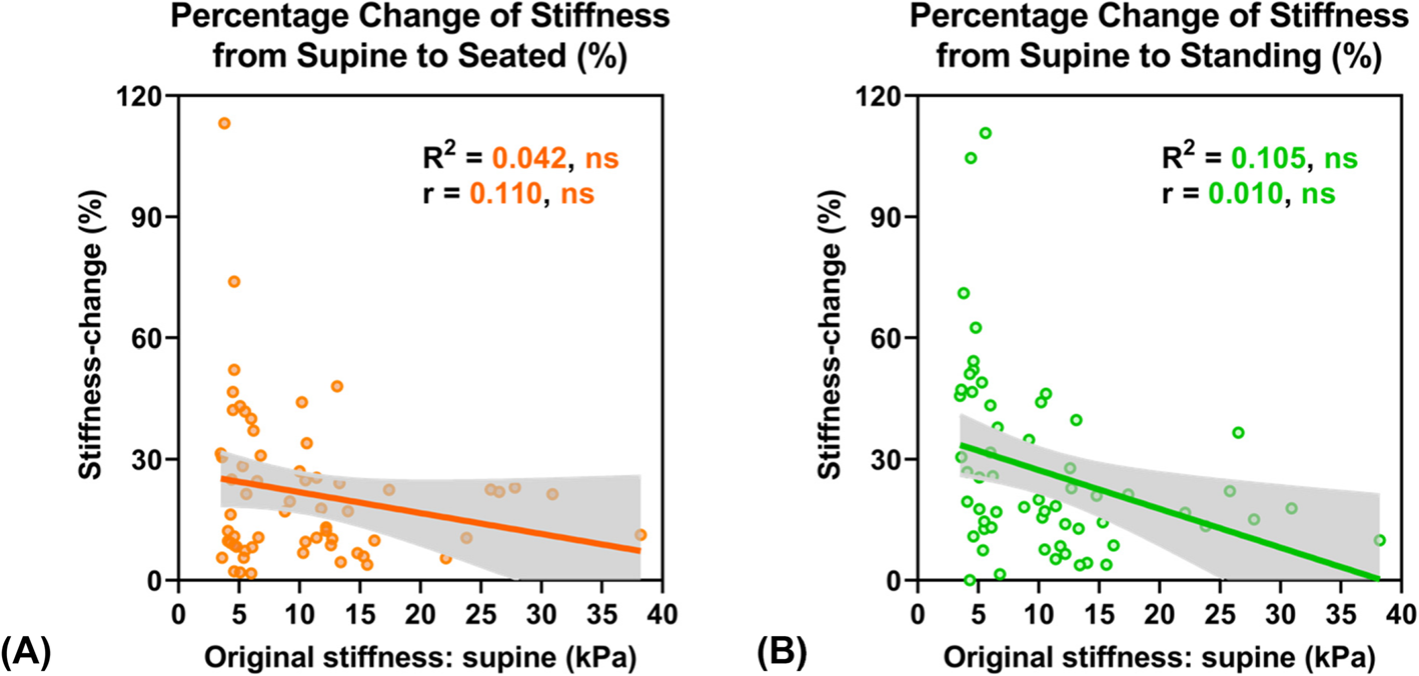
Relationship between LS measured in the supine posture (kPa) vs. magnitude of stiffness-change (%) when transitioning from the supine to (**A**) seated and (**B**) standing postures

Figure 4 depicts the absolute magnitude of stiffness-change in response to both seated and standing postures among 62 subjects. The Spearman correlation analyses showed a non-significant relationship between the LS measured in the supine posture and the magnitude of stiffness-change for either the seated or standing condition (both *p* > 0.05). No statistically significant association between these two variables implies that the ability of LS to change in response to an upright posture does not vary with fibrosis severity. Additionally, no statistically significant linear fit was observed between these two variables (both *p* > 0.05).

##### Inter-posture LSM quality criteria difference comparison

**Fig. 5:**
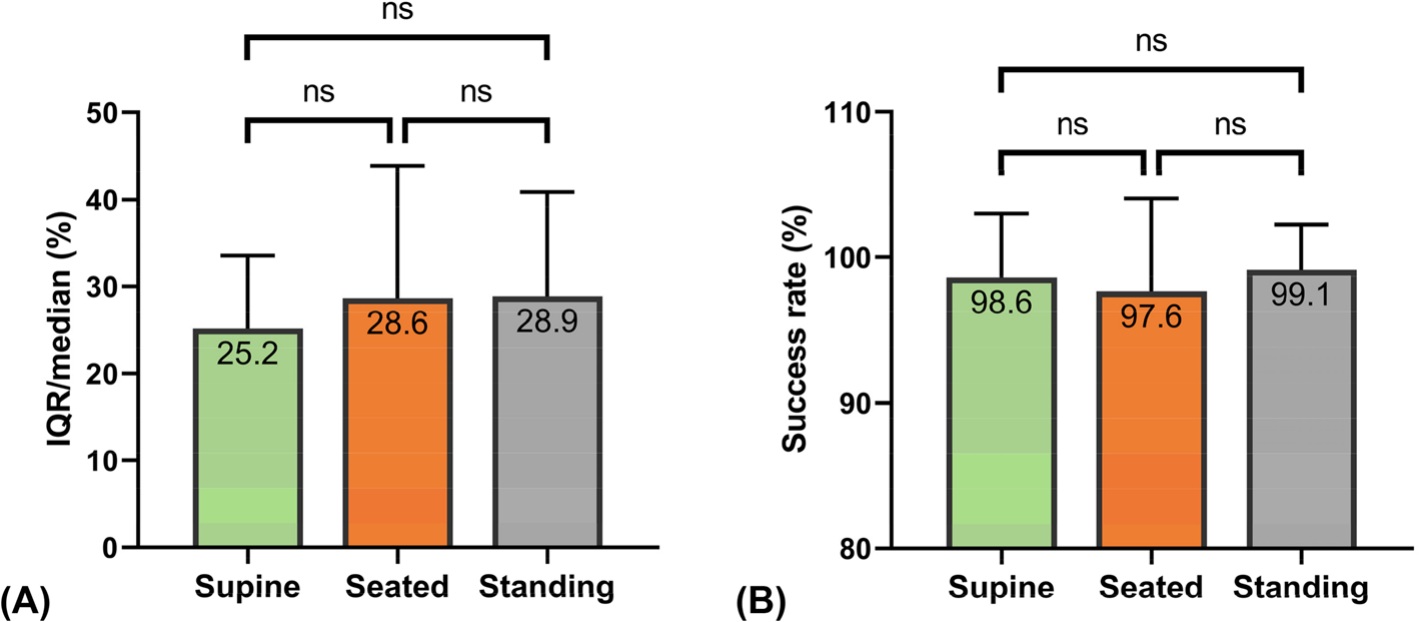
Effect of posture on the quality criteria of LSM: (**A**) IQR/median (%) of 10 LSMs; (**B**) success rate (%) of 15 LSM attempts (ns: *p* > 0.05; *p* ≤ 0.05 otherwise)

We also compared the feasibility and reliability of LSM among all posture conditions. Figure 5 depicts the success rate and IQR/median of LSMs by posture. Although the use of the upright position (seated, 29 ± 15%; standing, 29 ± 12%) marginally increased the IQR/median compared to the supine position (25 ± 8%), the differences were not statistically significant, *F*(2,111) = 2.233, *p* = 0.117, partial η^2^ = 0.04. Similarly, the incidence of successful LSMs did not significantly differ between different postures (supine, 98.6 ± 4%; seated, 97.6 ± 6%; standing, 99.1 ± 3%), χ^2^(2) = 2.710, *p* = 0.258.

##### Factors affecting LS

Tables 2 and 3 reveal the subject characteristics independently associated with LS under each of posture conditions. In univariate analysis (Table 2), weight, SCD, and WC were positively correlated to LS across all three postures (r_s_ = 0.26–0.47, all *p* < 0.05). BMI was significantly associated with LS in the supine and seated positions, but not in the standing position (*p* = 0.086). Interestingly, younger age fairly associated with greater LS value measured in the standing position (r_s_ = –0.270, *p* = 0.034). Increased LS was more likely identified in overweight or obese individuals. A history of biopsy-confirmed fibrosis was a significant determinant of LS regardless of body positioning. LS was not related to steatosis across postures (*p* = 0.767–0.849), as reflected by non-significant correlation with CAP scores (*p* = 0.154–0.648). In multivariate analysis **(**Table 3**)**, BMI and a history of biopsy-proven liver fibrosis remained independent factors associated with LS across all postures. Other factors, such as age and presence of Type 2 DM, no longer influenced LS after adjusting for fibrosis history.

**Table 2:** Factors associated with LS measured under three posture conditions in univariate analyses

**Characteristics**	**Supine LSM**		**Seated LSM**		**Standing LSM**	
	**r**_**s**_	***p***	**r**_**s**_	***p***	**r**_**s**_	***p***
Age (per year)	-	0.104	-	0.106	–0.270	0.034*
Weight (kg)	0.410	< 0.001*	0.308	0.015*	0.322	0.011*
BMI (kg/m^2^)	0.425	< 0.001*	0.268	0.035*	-	0.086
SCD (mm)	0.370	0.003*	0.277	0.029*	0.260	0.041*
WC (cm)	0.467	< 0.001*	0.369	0.003*	0.345	0.006*
CAP value (dB/m)	-	0.154	-	0.624	-	0.648
	**Median [IQR]**	***p***	**Median [IQR]**	***p***	**Median [IQR]**	***p***
Steatosis	Absent, 6.5 [11]; Present, 9.2 [7]	0.849	Absent, 7.8 [10]; Present, 7.3 [6]	0.877	Absent, 7.2 [10]; Present, 7.9 [6]	0.767
Sex	Male, 10.2 [10]; Female, 6.3 [8]	0.193	Male, 8.3 [9]; Female, 7.0 [6]	0.127	Male, 9.2 [9]; Female, 6.6 [6]	0.066
Overweight / obesity	Absent, 5.5 [6]; Present, 12.2 [5]	< 0.001*	Absent, 6.8 [4]; Present, 11.5 [7]	0.008*	Absent, 6.7 [4]; Present, 12.0 [7]	0.017*
Hypertension	Absent, 6.2 [9]; Present, 10.6 [9]	0.187	Absent, 7.3 [8]; Present, 8.0 [10]	0.464	Absent, 7.9 [8]; Present, 9.5 [9]	0.330
Type 2 DM	Absent, 5.8 [8]; Present, 11.8 [5]	0.013*	Absent, 6.9 [7]; Present, 11.1 [7]	0.032*	Absent, 7.1 [7]; Present, 11.3 [7]	0.111
Dyslipidaemia	Absent, 6.2 [9]; Present, 10.2 [7]	0.627	Absent, 7.2 [9]; Present, 8.0 [7]	0.527	Absent, 7.9 [7]; Present, 9 [7]	0.889
Metabolic syndrome	Absent, 6.2 [9]; Present, 11.0 [9]	0.119	Absent, 7.3 [7]; Present, 10.5 [7]	0.128	Absent, 7.5 [7]; Present, 9.9 [9]	0.293
Biopsy-proven fibrosis	No, 4.6 [1]; Yes, 13.1 [7]	< 0.001*	No, 5.1 [2]; Yes, 12.7 [7]	< 0.001*	No, 6.2 [2]; Yes, 13.0 [7]	< 0.001*

Age, weight, BMI, SCD, WC waist circumference were analysed by Spearman's correlation test; sex, overweight / obesity, presence of steatosis, hypertension, DM, dyslipidaemia, metabolic syndrome, and a history of biopsy-proven liver fibrosis were analysed by unpaired t-test or Mann–Whitney U test, where appropriate

^*^ *p* ≤ 0.05

*BMI* Body mass index, *SCD* Skin-liver capsule distance, *DM* Diabetes mellitus, *WC* Wwaist circumference, *CAP* Controlled attenuation parameter, r_s_ Spearman's rank correlation coefficient

**Table 3:** Factors associated with LS measured under three posture conditions in multivariate analyses

Characteristics	Supine LSM		Seated LSM		Standing LSM	
	**β**	***p***	**β**	***p***	**β**	***p***
Age (per year)	0.155	0.111	0.168	0.102	0.119	0.267
Weight (kg)	0.222	0.241	0.256	0.199	0.295	0.159
BMI (kg/m^2^)	–0.455	0.028*	–0.523	0.017*	–0.509	0.026*
SCD (mm)	–0.137	0.257	–0.154	0.227	–0.121	0.365
WC (cm)	0.345	0.094	0.415	0.057	0.429	0.060
Overweight / obesity	–0.042	0.784	–0.111	0.496	–0.155	0.367
Type 2 DM	–0.051	0.601	–0.028	0.786	–0.065	0.542
Biopsy-proven fibrosis	0.826	< 0.001*	0.791	< 0.001*	0.742	< 0.001*

Age, weight, BMI, SCD, waist circumference, overweight / obesity, DM and a history of biopsy-proven liver fibrosis were analysed by multiple linear regression, using a standard enter method

^*^ *p* ≤ 0.05

*BMI* Body mass index, *SCD* Skin-liver capsule distance, *DM* Diabetes mellitus, *WC* Waist circumference, *β* Standardized regression coefficient

## Discussion

LS can be influenced by factors beyond fibrosis stage, with body posture potentially serving as a clinically relevant confounder. This study employed a two-group comparison design to compare the change in LS under different body positions. Results indicate that the patient group of fibrotic or cirrhotic livers and the control group of healthy livers behaved very differently in response to an upright position. On the other hand, the feasibility and reliability of LSMs were not compromised upon postural changes. Our data have highlighted the fact that posture affected TE, which necessitates the standardization of patient positioning and advances the knowledge defining the physical parameter of LS.

### Posture effect on LS

To the authors’ knowledge, this is the first work to systematically report the effect of normal daily postures (i.e., supine, seated, standing) on LS in both healthy and diseased livers. Our data clearly demonstrate that, in comparison to the supine position, both seated and standing positions induced liver stiffening, resulting in increased LS among apparently healthy controls. Conversely, the two upright positions were associated with decreased LS in patients across a spectrum of fibrosis, implying a softening effect on the liver. Subgroup analysis revealed a marked decrease in LS during seating and standing in cirrhotic patients, whereas LS remained stable in patients with clinically significant fibrosis. The underlying mechanism explaining this posture dependency is rather complex. We speculate external respiratory pressure and internal hepatic hemodynamics as the contributors (refer to Supplementary Material for sound reasoning). Current literature on how LS would respond to a postural change is scarce. We only found three other previous studies investigating the relationship between posture and LS [31–33]. Adolf et al. first reported the potential confounding effect of posture in a congress abstract, noting a significant increase in LS in healthy individuals when seated or standing [32]. However, this study lacked ample statistical reporting, making the finding inconclusive. Another study based on point shear wave elastography (pSWE) further confirmed significantly higher LS in the standing position than in the supine position [33]. These prior results are consistent with our observations in the healthy livers. Nevertheless, little is known about the biomechanical response of fibrotic livers to postural changes. In a recent study, Suda et al. shed light on the differential effect of the supine vs. left lateral decubitus positions on LS [31]. Surprisingly, LS was found to be decreased by the left decubitus positioning in 17 cases with confirmed liver fibrosis. Although this study used a different technique, namely 2D-SWE, and involved a side-lying position, the observed liver softening aligns with our finding regarding the response to an upright position. According to our factor analyses, no significant effect of steatosis on the supine, seated, and standing LS was found. BMI was the only anthropometric covariate consistently influencing LS across all postures, even when adjusted for a history of histologically proven fibrosis. This finding is broadly in keeping with the literature [34–37]; high BMI is a well-established metabolic risk factor for increased LS, indicative of fibrosis progression.

### Posture effect on measurement success and reliability criterion

Prior to our work, whether postural changes would influence the feasibility and reliability of LSM is yet a question in the field. Our data suggest that adopting upright postures did not compromise the success rate and IQR/median, compared with the supine posture. The incidences of LSM failure remained below 2.5% across all three postures, which align with the 3.1% failure rate previously reported for the supine LSM in the largest feasibility study of TE [38]. This evidence further supports either seated or standing postures as the backup measurement protocol, especially in cases of repeated LSM failure in the supine posture. On the other hand, we observed relatively large variability between LSMs, as demonstrated by IQR/median (supine, 25 ± 8%; seated, 29 ± 15%; standing, 29 ± 12%). The inclusion of overweight individuals (35%) in our cohort, known as the main determinant of unreliable LSM [27], along with the use of a device with a small ultrasound beam width in this study [29], may account for this phenomenon.

### Practical values of upright positioning

Our study also demonstrated the feasibility of utilizing upright positioning for TE in liver fibrosis assessment. From a practical perspective, this new attempt offers greater operational flexibility and has several advantages. Traditionally, TE is performed in the supine posture. However, not all acutely or critically ill patients can lie comfortably in this posture, which also requires a bulky examination table. In this case, upright TE is of particular value for patients who cannot tolerate supine positioning, such as those with orthopnea, ankylosing spondylitis, or mobility constraints. Additionally, the literature widely recognized obesity as a predominant risk factor for TE failure, due to the obstruction of ultrasound and shear wave propagation by excessive subcutaneous tissues [38]. Our clinical observation indicated that upright TE may facilitate assessing those morbidly obese individuals. This is likely because the standing position tends to reduce overlying abdominal wall thickness due to gravitational forces, thereby minimizing subcutaneous fat interference and improving intercostal access. Future studies are necessary to specifically compare the success rate of supine vs. upright TE in obese populations. We further propose that due to its ease of implementation, the non-lying position could facilitate TE in special settings such as outpatient consultations, home visits, mass screenings, particularly when a supine setup is not readily available.

### Clinical relevance and implications

In the present study, we observed the divergent biomechanical responses to postural changes, which have implications for clinical decision-making. In our view, the strategy of assessing LS in different body positions has the potential to aid in the differential diagnosis of abnormally elevated LS. Specifically, liver softening during seating or standing could serve as an indicator to confirm the presence of fibrotic accumulation, while liver stiffening possibly suggests no fibrosis. Future research should consider incorporating this novel indicator into a TE-based algorithm to enhance the identification of patients at risk of liver fibrosis. Larger studies are warranted to further validate its diagnostic performance and compare it with the accuracy of TE alone. Additionally, our findings may add insight to why LS has been shown to be higher with older age [39]. Evidence suggests that the average American spends the two-thirds of the day in either the seated or standing postures [40]. In this context, longstanding LS elevation resulting from prolonged periods of upright postures over a lifetime and subsequent activation of fibrogenesis pathways [10, 11] are postulated to play a role, although more data are required.

TE has gained increasing clinical attention and is now incorporated into HBV treatment guidelines [3]. Therefore, LS results need to be interpreted cautiously in the context, as it presents significant consequences for patient management. According to our intraindividual analysis, discordance of at least one fibrosis stage was observed in 18 subjects (29%) following a postural change. These resultant false positive or negative cases would become a source of misclassification. Moreover, to prevent unnecessary treatment prescriptions, clinical practitioners must be aware of the posture factor potentially interfering with LS independent of the actual stage. Current clinical guidelines establish LSM cutoff criteria based solely on a supine setup [4–7]. Our study also emphasizes the need for standardizing patient positioning to ensure accurate TE assessment.

### Strengths and limitations

The strengths of this study include the use of several strategies to control for extraneous variables. For example, this study involved a repeated measures design effectively controlling interindividual differences, and patients were carefully evaluated to exclude other known confounding factors influencing LS before inclusion in the experiment. We further acknowledge the crucial role of real-time B-mode guidance in locating the liver and select a consistent intra-hepatic measurement location across postures, which significantly contributed to eliminating liver heterogeneity as a source of bias. Otherwise, abdominal organ relocation following a posture change will pose a challenge for conventional TE without sufficient visual guidance [31, 41]. Second, efforts were made to maximize the validity of LS. They included the inter-technique comparison and elastogram quality assessment, as supported by the results of strong correlation between conventional vs. B-mode guided TE and excellent inter-rater agreement, respectively (See Supplementary Fig. 1). Third, we sought to determine whether LSM would remain feasible and reliable in an upright position. Our data is a first-of-its-kind literature source for TE, and can inform the future uptake of this new positioning technique in clinical practice.

However, this study also has the following limitations. As a pilot proof-of-principle study, we included a relatively small cohort of 62 subjects. Although a *priori* calculation justified the adequacy of this sample size for the two-way mixed ANOVA, larger samples are preferable for additional subgroup analysis to increase greater statistical power. Furthermore, no participant was subjected to liver biopsy which is the reference standard for staging fibrosis. While being justified by ethical considerations, the fact merits further research attention to assess the relative accuracy of LS obtained from different postures against histology, and to determine which posture best represents the actual fibrosis state. Further factor analysis of the interaction between posture and various histological parameters on LSM will also become feasible. Additionally, we did not demonstrate the reversibility of LS between two posture sessions. It also remains unknown how long it takes for a change in LS to occur when transitioning from one posture to another. This data could inform the minimum duration of a rest period in the supine position required for measuring a stabilized LS. Such standardization efforts will be beneficial for everyday TE practice.

## Conclusions

We here studied LS in response to postural changes from the supine to upright positions. Our work provides definitive evidence that performing TE in the non-lying positions is feasible, at least with a B-mode image guided TE device. LS depends directly upon body posture, and its response to the upright position varies according to fibrosis stage. Although not completely understood, the observed dependency of LS on posture is explained by the hypothesized mechanism combining external and internal pressure sources. Our findings further unravel a previously unrecognized role of transitioning between different postures in the confirmatory diagnosis of liver cirrhosis. Additionally, postural changes show no significant effect on both the success rate and reliability of LSMs when using a B-mode image guided TE device. This has implications for clinical use of either seated or standing position as an alternative posture, especially in cases where obtaining LSMs proves challenging in the traditional supine position. However, caution should be exercised when interpreting LS obtained under different postures.

## Supplementary Information

Supplementary Material 1.

## Data Availability

The dataset that support the findings of this study are available on reasonable request from the corresponding author. The data are not publicly available due to privacy or ethical restrictions.

## Abbreviations

2D-SWE: Two-dimensional shear wave elastography
BMI: Body mass index
CHB: Chronic hepatitis B
CLD: Chronic liver disease
HBV: Hepatitis B virus;
IQR: Interquartile range
IVC: Inferior vena cava
LS: Liver stiffness
LSM: Liver stiffness measurement
MASLD: Metabolic dysfunction-associated steatotic liver disease
pSWE: Point shear wave elastography
SCD: Skin–liver capsule distance
SPH: Sinusoidal pressure hypothesis
TE: Transient elastography
