# Supplementary material for: Body posture can modulate liver stiffness measured by transient elastography: a prospective observational study
Source: BMC Gastroenterol. 2024 Oct 31;24:386. doi: 10.1186/s12876-024-03473-8 (PMC11526721; doi:10.1186/s12876-024-03473-8)
Supplement: Supplementary file 1 — Supplementary Material 1. [file 12876_2024_3473_MOESM1_ESM.docx]

# Supplementary Material

### ***Supplementary Methods***

**Attempts of experimental confounder control.** Considering that the liver is a moving organ and not necessarily uniform, measures were taken in an attempt to limit the confounding factors, i.e., respiratory motion and liver fibrosis heterogeneity. To control for the confounder of respiration, subjects wore a Go Direct^®^ respiration chest belt (Vernier, Beaverton, USA) sensing and monitoring respiratory cycles in real-time. LSMs were standardized at the end-inspiratory phase across postures, as suggested by previous studies for improved predictive accuracy and success rate of TE (1). To control for the confounder of heterogeneous fibrosis, upright LSM was performed near the initially identified site. Using the captured B-mode images as additional reference, the investigator located the same liver lobe of interest. LS was then obtained with reference to the exact location or landmark within the liver, from which the baseline supine LSM was made.

To validate the performance of B-mode guided TE, conventional TE using the widely validated Fibroscan^®^ system (Echosens, Paris, France) was administered to each subject on the same day. Following the previously detailed protocol(2, 3), a minimum of 10 valid acquisitions were made solely in the supine position for benchmark comparison.

**Determination of elastogram quality.** To calculate the success rate, elastogram acquisition quality was qualitatively analyzed by a human rater and determined on a binary scale. Manual elastogram classification primarily incorporates two criteria: the presence and quality of the shear wave propagation trajectory in an elastogram. Specifically, a rater utilized the dichotomy (i.e., presence or absence) to determine whether a shear wave trajectory was detectable and of sufficient quality for LS computation. A successful LSM was indicated by a high-quality elastogram, whereas a poor-quality elastogram invalidated LSM, potentially compromising the diagnostic accuracy. In this study, two raters, who had six years and six months of experience in interpreting elastogram respectively, independently classified all elastograms. They were blinded to each other's classification results and the actual posture condition of the elastogram being judged. In case of discrepancies between raters, a consensus classification result was assigned for subsequent analysis of inter-posture success rate comparison. Ultimately, the median LS (in kPa), alongside another two measures of IQR/median (%) and success rate (%), were reported for each posture condition.

**Statistical analyses.**

LS measured by conventional TE and B-mode guided TE in different positions were compared pairwise, and Spearman’s rank correlation coefficients (*r*_s_) were computed.

In a secondary analysis of rater agreement, we examined inter-rater percentage agreement and corresponding Cohen’s kappa (κ) for two raters who participated in classification of successful elastogram acquisition. The strength of agreement was interpreted based on Landis & Koch’s criteria (4): poor to fair (κ < 0.4), moderate (κ = 0.4–0.6), substantial (κ = 0.6–0.8), and excellent (κ > 0.8).

### ***Supplementary Results***

**Subject characteristics.** Compared with liver disease patients, controls were less overweight (proportion, 61% vs. 10%; p < 0.001) as indicated by a lower BMI (25 kg/m2 vs. 23 kg/m2; p < 0.001), skin–liver capsule distance (16 mm vs. 11 mm; p < 0.001) and waist circumference (96 mm vs. 81 mm; p < 0.001), but gender distribution (males, 71% vs. 52%; p = 0.192) and age (50 years vs. 58 years; p = 0.051) did not differ between the groups. In addition, subgroup analyses by posture (supine vs. seated vs. standing) revealed no differences in IQR/median between the groups. The success rate was not significantly different between the groups, except for those LSMs performed in the supine condition (100% for patients vs. 97% for controls; p = 0.005). In contrast, LS was significantly higher in the patient group than in the control group, consistently across the three postures (all p < 0.001).

**Inter-rater agreement.** The percentage agreement for the two raters was 99.5%, based on their assessments of whether the 2790 elastograms, obtained from three postures of all 62 study participants, were classified as successful acquisitions. Cohen’s kappa (κ) statistic was calculated to quantify the agreement between the novice and experienced raters’ judgements, yielding an excellent level of inter-rater agreement (κ = 0.813, 95% CI: 0.717–0.909, *p* < 0.005).

**Pairwise comparison of TE techniques.** As shown in **Supplementary Figure 1**, the correlation strength of LS between conventional and B-mode guided TE varied by posture (*r*_s_ ranging from 0.79–0.94, all *p* < 0.001). The strongest correlation (*r*_s_ = 0.936, 95% CI: 0.894–0.961) and linearity (R^2^ = 0.964) were observed in the pair of the supine conditions. On subsequent subgroup analysis, the correlation between techniques was more pronounced among liver disease patients (*r*_s_ = 0.867) than controls (*r*_s_ = 0.620).

(A)
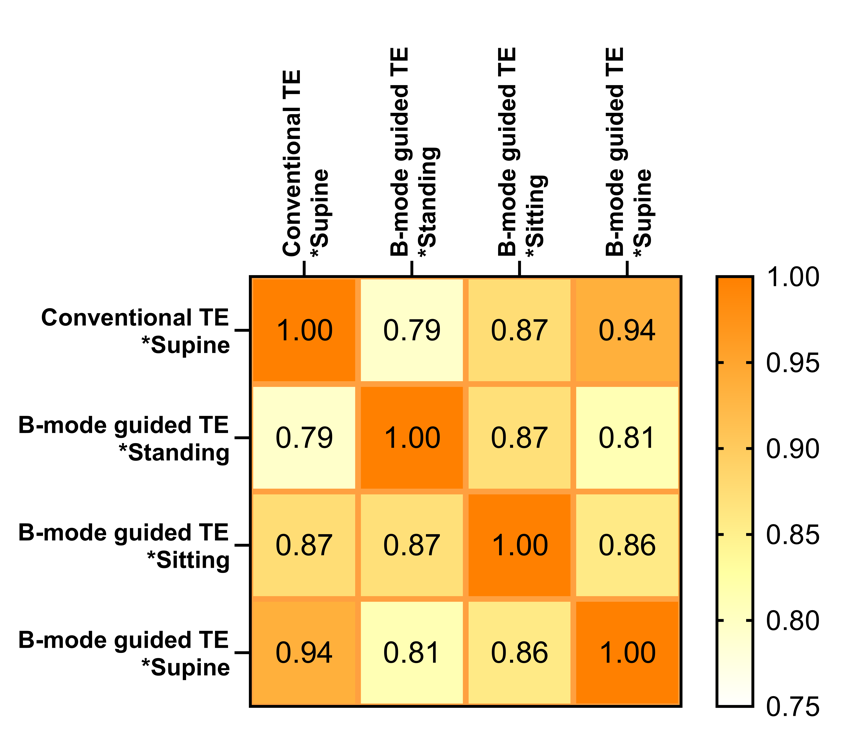


(B)**
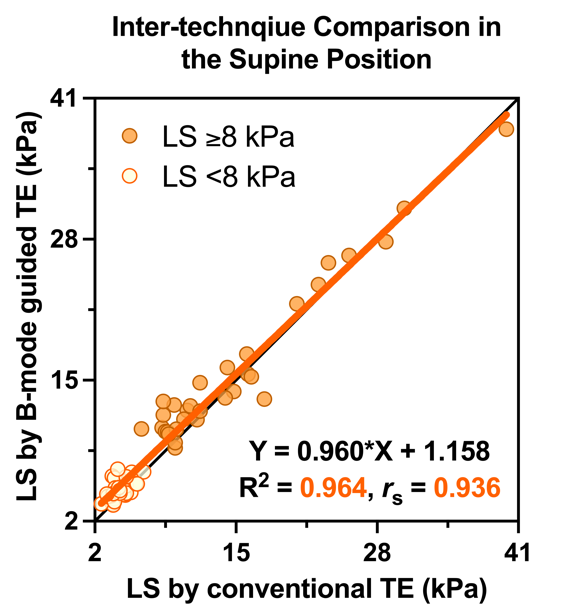
**

**Supplementary Figure 1.** Relationship between LS measured by conventional vs. B-mode guided TE: (A) spearman correlation matrix depicting LS comparison between TE techniques; (B) scatterplot with Spearman correlation and simple linear regression analyses (orange and light-yellow circles indicate the patients with liver diseases and the controls, respectively).

### ***Supplementary Discussion***

**Possible mechanism behind.** The underlying mechanism explaining the posture dependency on LS are rather complex, and remains a matter for speculation. The prevailing theory to explain relevant confounding conditions is the sinusoidal pressure hypothesis (SPH)(5), in which the pressure of hepatic sinus, reflecting LS, is affected by various pressure compartments. In this context, we initially hypothesized that physical interaction between the lung and liver would contribute to LS as a consequence of external pressure. Computational modelling indicated the morphological changes of the liver from the supine to seated position, notably a cranio-caudal translation of 21.9 mm due to the gravity (6). This shift resulted in less constraint applied on the liver during respiratory movement, thereby generally decreasing LS in the upright position. The impact of the lung–liver interaction was likely more pronounced in our cohort, as we performed end-inspiratory LSMs. Another influencing factor is hemodynamic changes in the liver, which provide internal pressure. An upright posture has been shown, across CT, MRI and ultrasound studies, to differentially impact the hepatic hemodynamics depending on the presence of fibrosis(7-9). In the healthy individuals, the IVC metrics such as the diameter (10), cross-sectional area (11) and flow velocity (11) decreased significantly from the supine to upright positions, leading to an outflow blockage with higher hydrostatic pressure (8, 12). This possibly caused intrahepatic blood stasis, and stretched the distensible Glisson’ capsule, which in turn increased LS. Conversely, the cirrhotic liver and IVC might be less deformed by an upright posture change (6, 8), which contributes to maintain stable venous drainage and resist dramatic changes in LS. Other factors like the uneven impact of gravity, intra-abdominal pressure differences, and delayed hemodynamic responses in cirrhosis might also play a role (8, 13, 14). Additionally, the tendency toward no difference between LS measured in the seated and standing postures is likely attributable to their similar hemodynamic characteristics. In summary, external respiratory pressure may synergistically act with internal hepatic hemodynamics to produce the diverse responses of LS observed in our study.

### ***Supplementary References***

1. Yun MH, Seo YS, Kang HS, Lee KG, Kim JH, An H, et al. The effect of the respiratory cycle on liver stiffness values as measured by transient elastography. Journal of viral hepatitis. 2011;18(9):631-6.

2. Shiha G, Ibrahim A, Helmy A, Sarin SK, Omata M, Kumar A, et al. Asian-Pacific Association for the Study of the Liver (APASL) consensus guidelines on invasive and non-invasive assessment of hepatic fibrosis: a 2016 update. Hepatology international. 2017;11(1):1-30.

3. Sandrin L, Fourquet B, Hasquenoph J-M, Yon S, Fournier C, Mal F, et al. Transient elastography: a new noninvasive method for assessment of hepatic fibrosis. Ultrasound in medicine & biology. 2003;29(12):1705-13.

4. Landis JR, Koch GG. The Measurement of Observer Agreement for Categorical Data. Biometrics. 1977;33(1):159-74.

5. Mueller S. Does pressure cause liver cirrhosis? The sinusoidal pressure hypothesis. World journal of gastroenterology : WJG. 2016;22(48):10482-501.

6. Hayes AR, Gayzik FS, Moreno DP, Martin RS, Stitzel JD. Comparison of Organ Location, Morphology, and Rib Coverage of a Midsized Male in the Supine and Seated Positions. Computational and mathematical methods in medicine. 2013;2013:419821-12.

7. Ohnishi K, Saito M, Nakayama T, Iida S, Nomura F, Koen H, et al. Portal venous hemodynamics in chronic liver disease: effects of posture change and exercise. Radiology. 1985;155(3):757-61.

8. Suda T, Sugimoto A, Kanefuji T, Abe A, Yokoo T, Hoshi T, et al. Gravity assistance enables liver stiffness measurements to detect liver fibrosis under congestive circumstances. World journal of hepatology. 2022;14(4):778-90.

9. Van Beers BE, Leconte I, Materne R, Smith AM, Jamart J, Horsmans Y. Hepatic Perfusion Parameters in Chronic Liver Disease: Dynamic CT Measurements Correlated with Disease Severity. American journal of roentgenology (1976). 2001;176(3):667-73.

10. Ishizaki Y, Fukuoka H, Ishizaki T, Kino M, Higashino H, Ueda N, et al. Measurement of inferior vena cava diameter for evaluation of venous return in subjects on day 10 of a bed-rest experiment. Journal of applied physiology (1985). 2004;96(6):2179-86.

11. Kadoya Y, Miyati T, Kobayashi S, Ohno N, Gabata T. Evaluation of gravity effect on inferior vena cava and abdominal aortic flow using multi-posture MRI. Acta radiologica (1987). 2021;62(8):1122-8.

12. Goertz RS, Egger C, Neurath MF, Strobel D. Impact of food intake, ultrasound transducer, breathing maneuvers and body position on acoustic radiation force impulse (ARFI) elastometry of the liver. Ultraschall Med. 2012;33(4):380-5.

13. Adolf S, Millonig G, Friedrich S, Seitz HK, Mueller S. 411 VALSALVA AND ORTHOSTATIC MANEUVERS INCREASE LIVER STIFFNESS (FIBROSCAN®) IN HEALTHY VOLUNTEERS. Journal of hepatology. 2010;52:S168-S.

14. Mederacke I, Wursthorn K, Kirschner J, Rifai K, Manns MP, Wedemeyer H, et al. Food intake increases liver stiffness in patients with chronic or resolved hepatitis C virus infection. Liver international. 2009;29(10):1500-6.
